# A guide to large-scale RNA sample preparation

**DOI:** 10.1007/s00216-018-0943-8

**Published:** 2018-03-15

**Authors:** Lorenzo Baronti, Hampus Karlsson, Maja Marušič, Katja Petzold

**Affiliations:** Department of Medical Biochemistry and Biophysics, Karolinska Institutet, Scheeles Väg 2, 17177 Stockholm, Sweden

**Keywords:** RNA, Sample preparation, In vitro transcription, Chemical synthesis, Preparative high-performance liquid chromatography, Structural biology

## Abstract

RNA is becoming more important as an increasing number of functions, both regulatory and enzymatic, are being discovered on a daily basis. As the RNA boom has just begun, most techniques are still in development and changes occur frequently. To understand RNA functions, revealing the structure of RNA is of utmost importance, which requires sample preparation. We review the latest methods to produce and purify a variation of RNA molecules for different purposes with the main focus on structural biology and biophysics. We present a guide aimed at identifying the most suitable method for your RNA and your biological question and highlighting the advantages of different methods.

**Graphical abstract:**
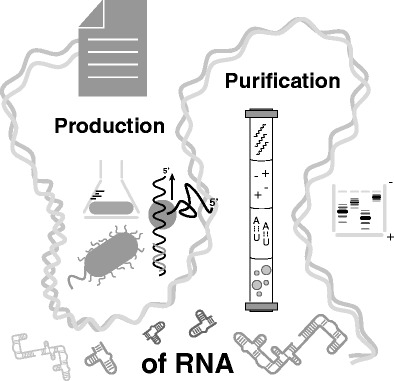
In this review we present different methods for large-scale production and purification of RNAs for structural and biophysical studies

In this review we present different methods for large-scale production and purification of RNAs for structural and biophysical studies

## Introduction

**Fig. 1:**
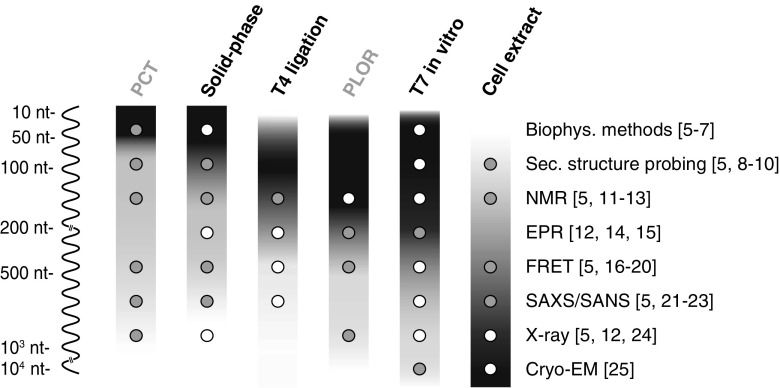
Overview of commonly used sample preparation methods for structural characterization of RNA. The size of RNAs that can be obtained is indicated by gradients for each of the methods ranging from black, for the most suitable, to white, indicating not applicable. White dots indicate which RNA sample preparation method is commonly used and well established for the specific structural biology method, whereas gray dots indicate a less common application or an upcoming new method. Preparation methods that were recently developed and are yet to show their full potential are written in gray above the gradients (Table 1). References for relevant examples are indicated to the right of each structural biology method. Abbreviations: biophys. biophysical, cryo-EM cryo-electron microscopy, EPR electron paramagnetic resonance, FRET fluorescence/Förster resonance energy transfer, NMR nuclear magnetic resonance, nt nucleotide, PCT polymerase chain transcription, PLOR position-selective labeling of RNA, SANS small-angle neutron scattering, SAXS small-angle X-ray scattering, sec. secondary

In an ever-growing world of new classes of RNAs, the need to reveal their function and structure is expanding [1]. This need coincides with the advancement in structural biology methods, such as the resolution revolution in cryo-electron microscopy (cryo-EM) [2] or the discovery of invisible RNA states by nuclear magnetic resonance (NMR) methods [3, 4]. Multiple techniques are now available to probe the features of a given RNA, and each one uniquely accesses structural information at different resolution, depending on the question posed. The required sample preparation on a large scale often constitutes the limiting step, as each structural method strongly relies on the quality (and often quantity) of the RNA sample that has to be provided (Fig. 1). Hence first we will give a short overview of the available techniques to determine RNA structure and dynamics.

X-ray crystallography produces high-resolution data but relies on RNA samples that are rigid enough to crystallize. It is often not limited by the size of the sample (Fig. 1) and is therefore commonly used to characterize large protein–RNA complexes. Cryo-EM has recently undergone a leap in enhancement of resolution that now rivals X-ray crystallography, especially for large and rigid molecules (Fig. 1). Whereas X-ray crystallography relies on crystals analyzed with high-energy synchrotron radiation, in cryo-EM, molecules are usually frozen on a surface and single molecules are observed with a high-resolution electron microscope. Both X-ray crystallography and cryo-EM have in recent years delivered detailed insights into ribosome structures [26, 27].

Fluorescence/Förster resonance energy transfer (FRET) is based on two fluorophore tags, which can transfer energy on the basis of their relative proximity. The distance between the tags can be assessed depending on the efficiency of the FRET exchange, and this information can then be used to calculate low-resolution structures. FRET structure calculation requires the tags be chemically bonded to the molecule, potentially inhibiting relevant interactions. Most often, more than one donor–acceptor pair is necessary, requiring several samples. However, this is a relatively fast method and usually requires a small amount of sample. FRET is most often used on ribosomes, but will likely have large use in the localization of regulatory RNA on genomic DNA or other RNA interactions [16].

Electron paramagnetic resonance (EPR) can be used to measure crude long-distance interactions on the basis of a spin label covalently bonded to the RNA of interest. The method is in its infancy but can provide information for challenging systems, where other structural methods fail. In analogy to FRET, it requires positioning of artificial spin labels that could potentially interfere with the native structure [28].

NMR methods provide atomic resolution information, allow secondary and tertiary structure determination, and make possible the characterization of molecular motion on a wide range of timescales [29, 30]. A broad range of sample conditions can be studied, from dilute solutions to in cell; however, RNAs larger than approximately 100 kDa are hardly detected by solution NMR methods [31]. ^15^N and ^13^C isotopic labeling of the sample is a prerequisite when high-resolution structural data need to be inferred from NMR. With increasing size, partial or total deuteration of the sample is required.

Small-angle X-ray scattering/neutron scattering measures the diffraction properties of the atoms in the sample and retrieves information on the envelope of the molecule. Purified samples can be measured in native solutions. Small-angle X-ray scattering/neutron scattering is often used as a complementary source of information for integrated structural biology studies as it can easily access the relative position of the components of large multimolecular complexes [32]. For small-angle neutron scattering, deuteration of the sample might be necessary.

Secondary structure chemical probing using enzymes or metal ions has been used for decades, but has recently seen a revival with small molecules such as selective 2′-hydroxyl acylation analyzed by primer extension (SHAPE) probing to reveal the RNA structure [33–35]. Structure determination by chemical probing is rather crude, and de novo calculation is still an exception. Information on secondary structure with nucleotide resolution is accessible when chemical probing is performed on purified or native samples in vitro or in vivo, respectively. It is applicable to large RNAs and protein–RNA complexes, and can be performed in a high-throughput fashion. Most protocols still require extraction and processing of the RNA of interest, and the experimental readout is based on sequencing or capillary electrophoresis, which can introduce their own biases.

Biophysical methods, such as UV spectroscopy, circular dichroism spectroscopy, and isothermal titration calorimetry, are routinely used as first characterization steps in structural biology approaches. They are normally performed fast and require a low amount of purified sample [36], however, they usually give only a single average signal over the whole sample of conformations.

Computational prediction is a valuable tool in RNA structural biology, and the field is fast growing with ever-improved software. To be able to validate the advancements in the field, the RNA structure prediction community have created the *RNA puzzle*, where different methods compete with and validate each other every 2 years, providing a great display of which software is available and how the different types perform [37].

All of these experimental methods need copious amounts of highly pure RNA to answer the different questions about RNA biology and structure. Unfortunately, working with RNA is hindered by one major hurdle, the ubiquitous presence of RNases, proteins that degrade RNA at a high rate. This requires careful and extensive preparation of laboratory equipment in a so-called RNase-free fashion [38] and/or the use of different, commercially available, RNase inhibitors.

This review aims to reach out to the inexperienced reader in the field of RNA structural biology and to provide a starting point for the sample preparation literature, including the latest innovations.

## Sample preparation

### Recombinant overexpression

Recombinant overexpression in *Escherichia coli* has revolutionized protein structural biology by allowing the production of large quantities of sample from cheap biomass fermentation and quick recovery from bacterial lysates with use of fusion tags for affinity purification. In contrast, the development of RNA heterologous expression has been heavily hampered by a few crucial factors; namely, the degradation by intracellular RNases, the large 3′-end and 5′-end heterogeneity of the transcripts, and the lack of efficient tags for affinity purification [39]. See for overview Table 1.

**Table 1:** Advantages and disadvantages of different RNA production methods to aid, in combination with Fig. 1, in the selection of the most suitable method

Method		Advantages	Disadvantages
Recombinant overexpression	tRNA scaffolds	Cost-effective, high yields	Cloning steps required, insert must not interfere with scaffold folding, extensive downstream purification
	Circular RNAs	Increased stability toward exonucleases	Not fully developed, extensive downstream purification
	Alternative hosts	Fast and easy purification from growth medium	Not fully developed, lower yield than *Escherichia coli*
	T7 in vitro transcription	Well-established method, fast, easy, and reproducible	Lower size limitation (construct >10 nt), yields and purity are construct dependent
Enzymatic methods	Ribozyme cleavage and T4 ligation	Allows segmental labeling, production of small constructs (<10 nt)	Multiple enzymatic and purification steps required, low yield
	PLOR	Allows segmental labeling, fast protocol	New technology, relies on a noncommercial robotic platform, low yield for multiple labels/modifications
	PCT	Exponential amplification of DNA template, cost-efficient, fast, and can incorporate modified nucleotides	New method, 1 reaction always produces 2 RNA fragments, construct sizes are limited (12 and 25 nt)
Chemical synthesis	Solid-phase chemical synthesis	Modifications possible, easy purification, fast, no sequence-specific optimization	Expensive equipment required, length limited to ~100 nt, limited availability and high price of labeled or modified phosphoramidites

*nt* nucleotides, *PCT* polymerase chain transcription, *PLOR* position-selective labeling of RNA, *tRNA* transfer RNA

#### Transfer RNA scaffolds

To circumvent such limitations, Ponchon and Dardel [39] developed a method that exploits transfer RNA (tRNA) as a protective scaffold to accommodate target sequences in the anticodon stem. A similar, although less popular, variant that uses 5S ribosomal RNA (rRNA) as a scaffold was also developed [40]. In the Ponchon and Dardel method, tRNA (human tRNA^Lys^_3_ or *E. coli* tRNA^Met^) chimeras are cloned into an expression vector, where the insertion of choice replaces the anticodon stem, while maintaining the native fold of the TΨC and D stem-loops. In this way the maturation machinery processes and correctly folds the chimeras to homogeneous species that stably accumulate in the host in up to 50 mg of RNA per liter of culture [39, 41]. The scaffold can then be cleaved, releasing the insert via a co-transcribed ribozyme, a DNAzyme, or by annealing of the construct with a pair of complementary DNA oligonucleotides and subsequent digestion of the undesired DNA/RNA hybrid with RNase H [39]. This technique has proven successful for a broad range of RNA sizes and structures, including the expression of fusion aptamers for affinity purification [39, 41]. General approaches to in vivo RNA expression have been extensively reviewed [42], including applications to biosensing [43] and production of small interfering RNA/microRNA therapeutics [44, 45]. A recent review focused on in vivo sample preparation for high-resolution structural studies presented the most efficient strategies to design, process, and separate scaffold RNA from the insert of interest [46]. Recombinant expression has also been applied to NMR studies of large RNAs (more than 40 nucleotides) where expensive atom-specific isotopically labeled nucleotides (^2^H, ^13^C, and ^15^N) are normally demanded for unambiguous data interpretation [11, 47, 48]. Recently, the Dayie group [49] showed that by combination of the tRNA scaffold with two metabolically different, complementary *E. coli* strains, it is possible to achieve cost-effective preparation of ^13^C selectively labeled NMR samples in vivo.

#### Circular RNAs

A new approach to increase sample stability from exonuclease degradation was proposed on the basis of circular RNAs, a novel class of RNAs characterized by the covalent 3′–5′ linkage found in many organisms. The pathways involved in generation of circular RNAs as well as in vitro methods for circularization were recently reviewed, including a potential heterologous RNA production [50]*.* Intronic circular RNAs generated during tRNA biogenesis in metazoans were also shown to be effectively used for recombinant RNA expression in vivo [51].

#### Alternative hosts for in vivo production

The possibility to use different expression hosts alternatively to *E. coli* is currently being explored (e.g., use of *Rhodovulum sulfidophilum* to produce recombinant human precursor miR-29b that is directly retrievable from the extracellular medium) [52, 53]. In a recent study, Pereira et al. [54] compared the performances of *E. coli* and *R. sulfidophilum*, showing that even though purification from intracellular fractions of *E. coli* produces a higher yield with shorter fermentation times, use of *R. sulfidophilum* as an expression host drastically simplifies downstream purification and limits protein contamination. Although far from being as broadly applicable as the tRNA scaffold in the *E. coli* method, the *R. sulfidophilum* approach highlights the potential behind noncanonical expression hosts for future in vivo RNA production.

### Enzymatic methods

RNA transcription is one of the three fundamental reactions that define the central dogma of biology, and the enzymes that perform such a reaction are ubiquitously expressed among all life forms. In most cases, RNA polymerases (RNAPs) have evolved to participate in multimolecular machineries that require different protein cofactors for each stage of RNA synthesis (initiation, elongation, and termination). Although possible, recapitulating transcription in vitro for most bacterial, archaeal, or eukaryotic systems is still challenging, and the yields are not suitable for biotechnological applications. In contrast, bacteriophages have evolved their RNAPs to optimize genome compactness and maximize transcription yields during the lytic phase of their life cycle. This results, in the case of bacteriophages T3, T7, and SP6, in RNAPs that comprise a single polypeptide chain and require only Mg^2+^ as a cofactor to perform highly processive RNA synthesis. These first observations [55] allowed the development of T7 in vitro transcription, now the most established method for enzymatic RNA preparation (Fig. 1).

#### T7 in vitro transcription

**Fig. 2:**
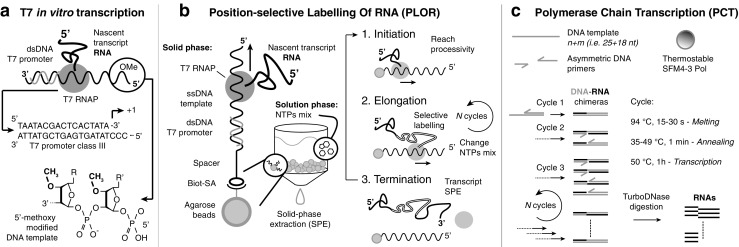
Transcription-based sample preparation methods. **a** T7 in vitro transcription scheme; the double-stranded (dsDNA) T7 promoter sequence and 5′-methoxy modification of the DNA template strand are highlighted. **b** Position-selective labeling of RNA in the solid and solution phase (left). Fundamental steps in one cycle that correspond the production of a single RNA transcript from one DNA template (right). **c** Polymerase chain transcription components (DNA template, asymmetric DNA primers, and SFM4-3 Pol) and reaction cycles. The RNA is exponentially amplified through thermal cycling transcription reactions; the DNA–RNA chimeras are subsequently digested to obtain the final RNA products. Abbreviations: biot biotin, nt nucleotide, NTP nucleotide triphosphate, RNAP RNA polymerase, SA streptavidin, ssDNA single-stranded DNA

In brief, T7 RNAP accepts ribonucleotide triphosphates as a substrate to synthesize a transcript RNA complementary to a DNA template of choice. Once the enzyme has completed the polymerization, it *runs off* the DNA template and releases the transcript, thereby ensuring the process is performed several times (Fig. 2a). In this way, milligram quantities of RNA can be produced per milliliter of transcription reaction in a few hours. Historically, two major drawbacks of the T7 system are promoter specificity and heterogeneous ends. Firstly, RNAP has a strong specificity for the promoter sequence, which extends beyond the initiation site, and therefore this limits the possibility to incorporate any user-defined 5′ sequence (Fig. 2a). Secondly, the inconsistent runoff of the RNAP leads to 3′-end inhomogeneity of the transcript, most commonly with a single A extension. To overcome such limitations, many improvements to T7 in vitro transcription have been made, and excellent overviews of transcription optimization techniques, including step-by-step protocols, are available [48, 56]. Recently, the combination of two previously known, yet unrelated, approaches to in vitro transcription was shown to greatly increase 3′-end homogeneity: the use of C2′-methoxy-modified DNA templates in the last two positions at the 5′ end of the DNA template reduces incorporation of 3′-end nontemplated nucleotides [57] (Fig. 2a), whereas low concentrations of dimethyl sulfoxide added in the transcription reaction increase T7 RNAP synthesis yield through an unknown mechanism [58]. Recent observations showed that C2′-methoxy-modified DNA templates in combination with 20% dimethyl sulfoxide reduce the addition of nontemplated nucleotides at the 3′ end to the extent that subsequent purification from side products was no longer necessary for the structural studies proposed in the study [59]. This approach was successfully applied to the transcription of the well-established 5′ leader of HIV genome RNA construct, where 3′-end inhomogeneity impairs the dimerization properties of the construct, thereby improving sample preparation and allowing a high-resolution NMR study of the whole intermolecular interaction site [60].

#### Ribozyme cleavage and T4 ligation

Complementary approaches to obtain 5′-end and 3′-end homogeneous RNAs use the nuclease activity of different enzymes to trim the transcript to the desired length. Cleavage of the phosphodiester backbone at a specific site is commonly achieved by design of a fusion transcript carrying a *cis*-acting, self-cleaving ribozyme [61, 62]. Most *cis*-acting ribozymes co-transcriptionally fold and cleave the backbone at a specific site with high efficiency and few or no sequence requirements. When expensive, labeled ribonucleotide triphosphates are used during transcription, *trans*-acting ribozymes can also be used after purification of the RNA of interest. Hammerhead, hepatitis delta virus, and Varkud satellite ribozymes are commonly used ribozymes, and their application, in particular to segmental labeling, was reported recently [48]. Despite their wide use, one major limit of hammerhead and hepatitis delta virus ribozymes is the generation of 5′-hydroxyl and 2′-3′-cyclic phosphate ends, which requires tedious additional sample treatment when native 5′-phosphate and 3′-hydroxyl ends are needed [63]. DNAzymes can also be used as *trans*-acting ribozymes; these, however, also generate noncanonical ends and require a purine/pyrimidine site for cleavage. Alternatively, canonical 5′-phosphate and 3′-hydroxyl ends can be obtained by RNase H cleavage after annealing of the target cleavage site with C2′-methoxy RNA/DNA chimera oligonucleotides. Recently, the development of new bioinformatic tools led to the discovery of a new class of ribozymes called “twisters” and comprising twister, twister sister, pistol, and hatchet [64, 65]. Structural characterization of twisters has begun to emerge, suggesting interesting potential for the future [66]. However, a general consensus on the mechanism of action of these new ribozymes is still lacking [67], and biotechnological application seems far away at the moment. 5′-Hydroxyl and 2′-3′-cyclic phosphate ends generated by ribozymes are a useful tool to avoid self-ligation and achieve the correct order of ligated fragments in segmental labeling protocols [48]. Combination of fragments with different labeling properties is a fundamental approach in the study of large RNAs (more than 40 nucleotides) by NMR experiments or for selective placement of paramagnetic and fluorescent probes for EPR and FRET experiments, respectively. Most segmental labeling techniques are based on sequential steps of enzymatic cleavage and ligation of in vitro transcribed or chemically synthesized RNA oligonucleotides. Ligation occurs between an acceptor fragment bearing a 3′-hydroxyl and a donor fragment with a 5′-monophosphate end. The ligation reaction can be performed with the T4 DNA ligase with the aid of a DNA splint complementary to the site of ligation (splint ligation) or by the T4 RNA ligase, which ligates donor and acceptor ends, which are brought close together by base pairing (nonsplinted ligation). A comprehensive review of available methods and detailed protocols of the most recent segmental labeling strategies proposed by the Allain group are available [48, 68]. Most of these techniques are affected by the inefficiency of T4 DNA ligase, and the final yield is normally low even for a two-fragment reaction. In the ideal scenario, where yield and downstream purification are optimized, the protocol can take up to 7 days to be completed [48, 68]. Moreover, when the fragments to be ligated are about the same size as the ribozyme used in the cleavage reaction, purification of the product of interest can be troublesome.

#### Position-selective labeling of RNA

An alternative to ligation methods for segmental labeling was recently developed by the Wang group [69], combining T7 in vitro transcription with the solid-phase synthesis approach. Position-selective labeling of RNA (PLOR) exploits the ability to pause and restart T7 transcription by supplementing the reaction mixture with incomplete nucleotide triphosphate (NTP) mixes in a stepwise fashion (Fig. 2b). In the original work, the authors synthesized the 71-nucleotide aptamer domain of an adenine riboswitch (riboA71) with selective labeling of the stem, linker, or loop regions. In this case the DNA template is incubated with T7 RNAP and an NTP mix (unlabeled) lacking CTP that causes stalling of the transcription at the position where the first CTP would be incorporated. Next, the DNA–RNAP–transcript tertiary complex bound to the solid phase is separated from reagents and cofactors in the liquid phase through extensive washing steps (Fig. 2b). The reaction is reinitiated by addition of a new NTP mix (labeled or unlabeled) that allows elongation of the transcript up to a new stalling site. Addition of incomplete NTP mixes can be repeated as many times as the nucleotide sequence allows for correct incorporation and stalling, and depending on the number of labeling sites wanted. Once the RNAP reaches the last stalling point, a full NTP mix is added to terminate the transcription. The transcripts are collected from the liquid phase, and reinitiation is inhibited by rapid cooling to 4 °C. The agarose-bound DNA template can be reused several times, and the whole sequence of initiation, elongation, and termination can be repeated for *N* cycles (Fig. 2b). Because of the many incubation/washing steps involved, a fully automated platform that allows fast and efficient PLOR synthesis was developed [69]. This approach has proven successful for the production of different samples of riboA71 for NMR studies, single molecule FRET studies [12, 69], and X-ray free electron laser serial crystallography.

#### Polymerase chain transcription

The iconic production of DNA via polymerase chain reaction amplifies exponentially the initial templates through thermal cycling reactions with high efficiency and minimal side products. Although in principle possible, RNA amplification via polymerase chain reaction has never been developed, mainly because of the lack of efficient heat-resistant RNAPs. A different approach was used by Cozens et al. [70], changing a DNA polymerase into an RNAP. To allow substrate tolerance of *Thermococcus gorgonarius* DNA polymerase, the group developed a mutant enzyme capable of synthesizing RNA and modified nucleic acids while retaining its stability toward heat denaturation [70]. The Romesberg group recently reported a variant of the Stoffel fragment of the *Taq* DNA polymerase (SFM4-3) [71] that was shown to exponentially amplify two different RNA fragments from one DNA template, primed by two asymmetric DNA oligonucleotides [5] (Fig. 2c). The reaction was named “polymerase chain transcription” and has the potential to exceed T7 transcription in terms of amplification levels (10^2^–10^4^-fold reported). It allows incorporation of 2′-F-modified NTPs and, because of the increased reaction temperature in comparison with in vitro transcription, allows a more efficient transcription of structured DNA templates with high GC content.

Although generally dominated by chemical synthesis, the development of engineered DNA polymerases for the synthesis of modified nucleic acids is a growing field, and enzymatic modification of NTPs and RNA oligonucleotides answers the need for unnatural oligonucleotides in synthetic biology, imaging, and therapeutics. A detailed description of the available modification methods is beyond the scope of this review; however, we direct the interested reader to a series of reviews that highlight the latest advancements in polymerase engineering and enzymatic synthesis of modified RNAs [72–76].

### Chemical synthesis

**Fig. 3:**
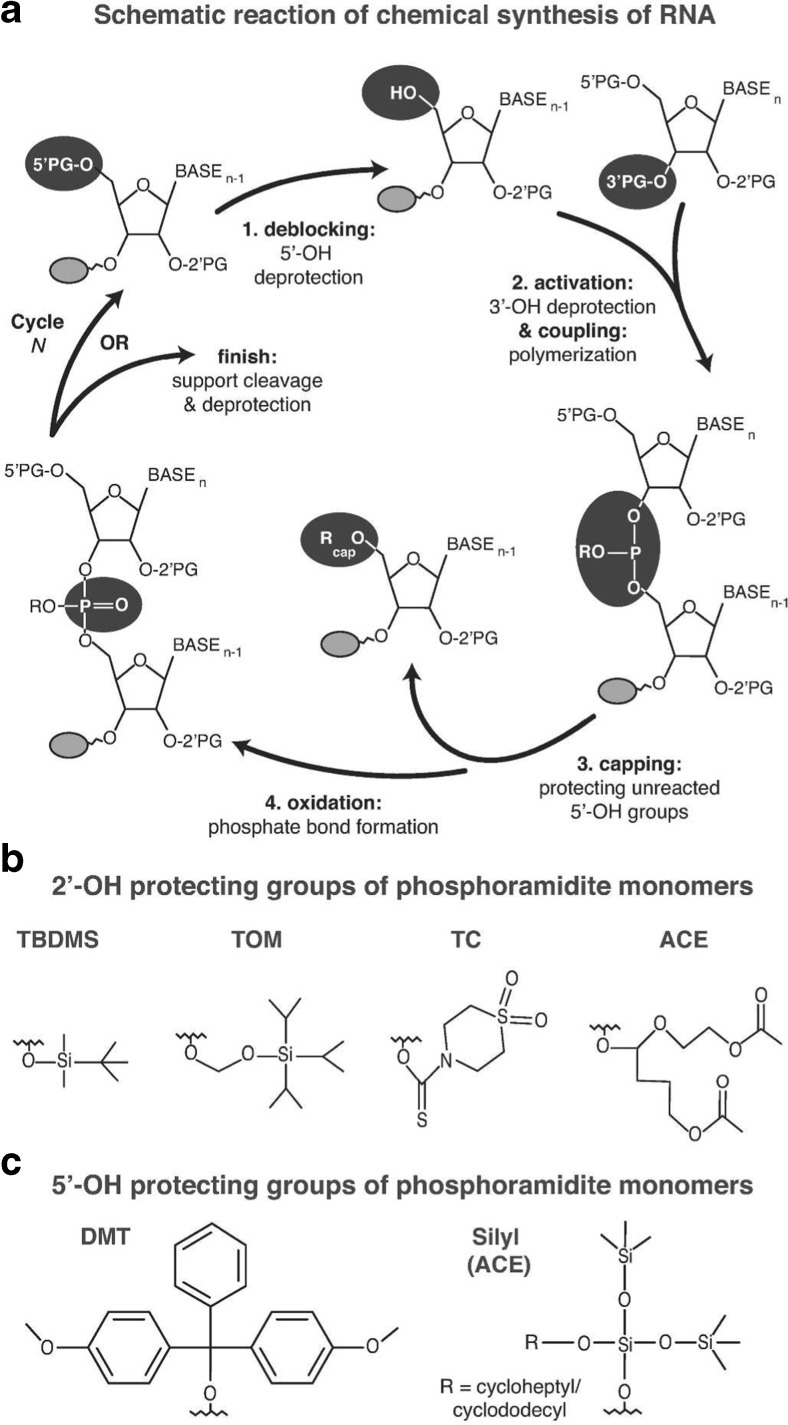
Solid-phase synthesis of RNA. **a** The different steps in the solid-phase synthesis cycle of RNA and the most commonly used. **b** 2′-OH and **c** 5′-OH protecting groups (PG). At the end of the production cycle, functional groups that are not participating in the polymerization reaction are deprotected. Abbreviations: ACE 2′-bis(2-acetoxyethoxy)methyl, DMT 4,4′-dimethoxytrityl, TBDMS 2′-*O*-(*t*-butyldimethylsilyl), TC 2′-thiomorpholine-4-carbothioate, TOM 2′-*O*-[(triisopropylsilyl)oxy]methyl

Currently, solid-phase chemical synthesis is the method of choice for the production of oligonucleotides shorter than 10 nucleotides, as these cannot be produced efficiently by the T7 system (Fig. 1) [77]. The upper size limit of the method is approximately 80 nucleotides. If longer constructs are desired, shorter chemically synthesized RNAs can be linked together with the help of T4 DNA or RNA ligase in the splint ligation systems [17]. Synthesis proceeds from the 3′ end to the 5′ end, and involves four steps (Fig. 3a). First, the 5′-hydroxyl protecting group of the nucleotide that is bound to the solid support is removed to allow the 5′-hydroxyl to be attacked by the activated 3′-hydroxyl group of the incoming nucleoside phosphoramidite monomer during the second, coupling step. In the third, capping step, unreacted 5′-hydroxyl groups are blocked from participating in subsequent reactions to avoid synthesis of side products. Finally, the unstable phosphite triester formed during the coupling step is converted to a stable species by an oxidation step, and the whole cycle can be repeated to obtain the desired length of the oligonucleotide polymer (Fig. 3a) [78]. Postsynthetic steps include cleavage from the solid support, which is either polystyrene or controlled-pore glass, and deprotection of reactive groups of the nucleobases. Purification of the whole-length product is in principle more straightforward than with enzymatic methods, as the most commonly used 5′-hydroxyl protecting group, 4,4′-dimethoxytrityl, can be left attached to the 5′-end nucleotide and used for purification of the whole-length product with reversed-phase high-performance liquid chromatography (HPLC).

The efficiency of solid-phase RNA synthesis depends on the type of RNA phoshoramidite monomers used, where currently four different RNA phosphoramidite monomers are commercially available. All use a mild base-labile protection on the amino groups of cytosine, guanine, and adenine nucleobases as well as on phosphate and the support linkage. Variations exist, however, with 5′-hydroxyl and 2′-hydroxyl group protection. 2′-*O*-(*t*-Butyldimethylsilyl) [79, 80] and 2′-*O*-[(triisopropylsilyl)oxy]methyl [81] groups are most commonly used for hydroxyl protection (Fig. 3b, c), and these allow the use of modifications that are sensitive to deprotection conditions. However, the biggest drawback of these moieties is long coupling times and the ability to synthesize only shorter (up to 60 nucleotides long) oligonucleotides. For longer RNAs, more expensive phosphoramidite monomers with shorter coupling times and greater coupling efficiencies can be used: 2′-bis(2-acetoxyethoxy)methyl monomers [82] and 2′-thiomorpholine-4-carbothioate monomers [83] (Fig. 3b, c). A main drawback of 2′-bis(2-acetoxyethoxy)methyl RNA monomers is the need to use fluoride to remove the 5′-hydroxyl protecting group, requiring modifications of current RNA synthesizers [78]. In contrast to all other types of phosphoramidite monomers, all 2′-thiomorpholine-4-carbothioate protecting groups can be removed in the same conditions, which makes RNA synthesis more convenient and less time-consuming. For a more detailed review of the current reactions used, see [78, 84].

Solid-phase chemical synthesis of RNA allows the introduction of isotopically labeled groups or chemical modifications at specific positions directly during the synthesis or through a postsynthetic labeling reaction with a reactive group [85, 86]. This is especially useful for techniques that require site-specific labeling schemes for simplification of spectra (NMR), need the incorporation of dyes and florescent probes (FRET), or require precise positioning of spin labels (EPR) (Fig. 1). The limitation for incorporation of modified and/or labeled residues in chemically synthesized RNA lies only in obtaining the appropriate reactive phosphoramidite monomers. A subset of monomers with chemically modified or fluorescent groups are commercially available, and several groups are working on new modified or labeled RNA building blocks that have been successfully incorporated with classical solid-phase synthesis [87–89]. Lately, improvements in isotope labeling of RNA, especially site-specific deuteration and segmental labeling of RNA, have opened the avenue for studying RNA molecules of ever-increasing size particularly by NMR spectroscopy [90]. For example, a novel strategy for resonance assignment that combines new strategic ^13^C labeling technologies with filter/edit-type nuclear Overhauser effect spectroscopy experiments to greatly reduce spectral complexity and crowding was proposed very recently [91]. This new strategy allowed assignment of important nonexchangeable resonances of proton and carbon nuclei with only one sample and less than 24 h of NMR instrument time for a 27-nucleotide-long model RNA. Another example includes the use of site-specific labeling of the ribose C1′ or C2′ position that allows measurements of dynamics with use of Carr–Purcell–Meiboom–Gill sequences [92].

## Purification methods

### Precipitation and solvent extraction

Selective isolation of RNA from complex mixtures is a step needed in most purification protocols to achieve a first, *rough* refinement of the sample before more sophisticated separation techniques. Precipitation and solvent extraction methods take advantage of differential solubility of biomacromolecules in different solvents and ionic conditions. Precipitation is generally performed after in vitro enzymatic reactions to separate the RNA of interest from the protein and DNA components or simply for buffer exchange, whereas solvent extraction followed by precipitation is the method of choice to isolate large amounts of total RNA from natural sources. See for overview Table 2.

**Table 2:** Advantages and disadvantages of different RNA purification methods

Method		Advantages	Disadvantages
Precipitation		Fast and cost-effective	Concentrated sample required, incomplete precipitation can occur
Solvent extraction		Established method	Toxic chemicals used in the protocol, low yield
Ultracentrifugation		Established method, simple	Only suitable for large macromolecular complexes and organelles
PAGE		Established method, applicable to a wide range of RNA sizes, cost-effective	Time-consuming, prone to RNase contamination. Final sample can contain contaminants
Liquid chromatography			
	RP-IP-HPLC	Analytical amounts: high resolution, multitude of established methods available	Columns limit loading capacity and resolution for preparative conditions, expensive chemicals used
	IE-HPLC	Native purification possible, low or high salt content of the elution buffer possible	Purified material can contain traces of elution salts, denaturing conditions require the use of toxic chemicals
	AC	Native purification possible, highly selective	Separation efficiency depends on the binding affinity between the tag and the ligand. Tags can interfere with downstream application, and their removal requires additional processing steps
	SEC	Native purification possible	Separation efficiency can be affected by alternative folding and the hydrodynamic radius of the molecule of interest

*AC* affinity chromatography, *HPLC* high-performance liquid chromatography, *IE* ion exchange, *IP* ion paring, *PAGE* polyacrylamide gel electrophoresis, *RP* reversed phase, SEC size-exclusion chromatography

#### Precipitation

The polar nature of the negatively charged backbone makes RNA highly soluble in water. Several cations used in combination with ice-cold ethanol as a co-solvent can effectively neutralize the backbone charges and reduce the solubility to a point where the RNA selectively precipitates out of solution. Different cations and their salts, such as ammonium acetate and lithium chloride, can be used depending on the size and concentration of the RNA to be precipitated. A comprehensive method comprising step-by-step protocols for RNA precipitation can be found in [93].

#### Solvent extraction

RNA isolation by acid guanidinium thiocyanate–phenol–chloroform extraction was initially developed as an alternative to the tedious total RNA isolation from mammalian tissues using ultracentrifugation. In this method the sample is incubated with an equimolar mix of phenol and chloroform, which allows proteins to be denatured by the guanidinium thiocyanate and consequently separated in the organic phase, whereas RNA is dissolved in the aqueous phase. Separation of the two phases is then achieved by centrifugation, and RNA can be retrieved by subsequent ethanol or lithium chloride precipitation. In acid guanidinium thiocyanate–phenol–chloroform the polar phase is kept under acidic conditions (pH 4–6), allowing the RNA to remain soluble while the DNA undergoes repartition at the interface. A detailed and reedited version of the original protocol was published [94], and many commercial kits based on this method are available.

### Ultracentrifugation

Ultracentrifugation is the standard way of purifying large RNA machines, such as ribosomes and ribosomal subunits, as well as other macromolecules of biological origin in the same size range. Ultracentrifugation with a gradient of a solute such as sucrose has been used for more than half a century [95]. For the isolation of complete ribosomes, polysomes, or individual ribosomal subunits, large amounts of cells from the organism of interest are mechanically lysed, the lysate is subsequently centrifuged at low speed to get rid of cell debris, and is then ultracentrifuged at high *g* (approximately 10^5^*g*) on top of a sucrose *cushion* to form a pellet of the required ribosomes [96]. The material can then be further purified by ultracentrifugation on a variety of buffered sucrose gradients with different salt content. The ability of the ribosome to keep its two subunits together is highly magnesium dependent, and by tuning the Mg^2+^ content of the sucrose gradient used for purification, one can obtain complete ribosomes or individual subunits. A lower magnesium ion concentration (1 mM) will aid in separating the individual subunits, whereas a higher concentration will promote isolation of complete ribosomes [97]. After separation of the ribosomal components, the UV absorbance of the content from the centrifugation tubes is measured, and the content is fractionated. From the absorbance profiles, the fraction content can be deduced [96]. Although traditionally heavily used within the field of structural biology in X-ray crystallography and cryo-EM, the ultracentrifugation technique has also found its way into other fields of research. Multiple methods where ribosomes are isolated for investigation of the messenger RNA being translated at a specific moment, the *translatome*, also use the ultracentrifugation technique [98, 99]. Ultracentrifugation has also proven useful for nucleic acid related nanotechnology or *DNA origami*, where ultracentrifugation onto glycerol gradients has been used as a good complement to more conventional and established agarose gel electrophoresis purification methods [100].

### Polyacrylamide gel electrophoresis

For decades, polyacrylamide gel electrophoresis (PAGE) was the standard method to purify large amounts of RNA (microgram to milligram scale) with single-nucleotide resolution as it can be easily applied to a wide range of RNA sizes and requires a minimal setup with cost-effective reagents. Like all electrophoretic techniques, PAGE uses a polymeric mesh to separate molecules by size, and/or conformation, as charged macromolecules migrate through an electric field. The mesh is prepared by polymerization of an acrylamide/bisacrylamide solution. The concentrations of acrylamide/bisacrylamide monomers can be varied to obtain the desired pore size and resolving power in the final mesh. Typical monomer concentrations range from 5% to 20% for RNA gels with 1:19 acrylamide/bisacrylamide relative ratio. In this range, short to intermediate length (5–500-nucleotide) RNAs can be resolved [101]. The acrylamide solution can be supplemented with a denaturing agent (most commonly 8 M urea, called “denaturing PAGE”) to fully unfold the migrating RNA and separate the molecules solely by size. With this approach, single-nucleotide resolution can be easily achieved in large-scale preparations, and it is a common analytical method. When no denaturing agent is used and the electrophoresis apparatus is externally cooled to prevent overheating caused by electric current flowing through the gel, conformers with different hydrodynamic radii of a given RNA construct can be resolved; this is also the underlying principle of electrophoretic mobility shift assays [102]. Isolation of the RNA of interest is obtained by excision of the band of interest from the gel, followed by electroelution or crush and soak extraction [103]. Gel excision is routinely used, and requires only a UV lamp to identify the band of interest by UV shadowing on fluorescent paper and a scalpel to excise the band. The excised band is then treated to allow diffusion of the RNA from the gel mesh into solution, and the RNA is subsequently purified by ethanol precipitation and resuspended in a buffer of choice. The impact of UV shadowing on RNA integrity has recently been addressed, revealing that commonly used lamps and exposure times can lead to minimal but detectable photodamage of the sample, and thereby potentially corrupt downstream structural studies [104]. Electroelution requires a dedicated apparatus that allows collection of the species corresponding to the band of interest being eluted from the gel pieces. RNA preparative PAGE has been used for decades, and its use has been well described in textbooks; however, a more comprehensive step-by-step protocol has recently been published with a focus on RNA preparation for structural biology application [105].

### Liquid chromatography

**Fig. 4:**
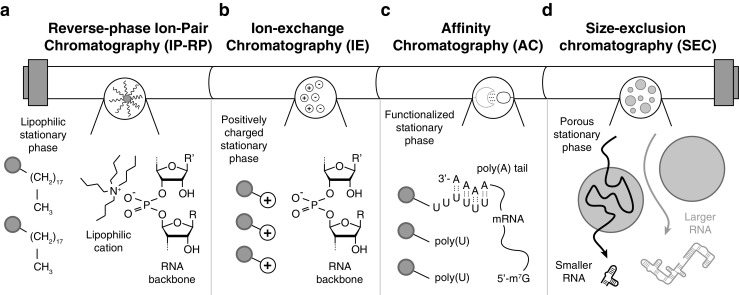
Liquid chromatography methods. **a** Reversed-phase ion-pairing chromatography; the lipophilic stationary phase retains the RNA thanks to a lipophilic cation-pairing agent (tetrabutylammonium is depicted). **b** Ion-exchange chromatography; a positively charged stationary phase interacts and retains the negatively charged RNA molecules. **c** Affinity chromatography; a polyuridine (poly(U)) functionalized stationary phase selectively interacts with polyadenosine (poly(A)) tails of messenger RNAs (mRNA). **d** Size-exclusion chromatography; large RNAs are eluted through the porous medium of the stationary phase with short retention times, and smaller RNAs are absorbed into the porous medium, resulting in longer retention times

Liquid chromatography techniques are well established for the purification and analysis of nucleic acids and oligonucleotides spanning a wide size range. On the basis of the concept of allowing solutes in a solvent (mobile phase) to run through a column containing a solid material (stationary phase) that interacts with the solutes to different extent, separation is achieved [106]. Here we focus on the chromatographic methods that are relevant for RNA sample preparation (Fig. 4). Among the sorptive separation techniques commonly used for nucleic acid separation are reversed-phase ion-pairing HPLC (RP-IP-HPLC), ion-exchange HPLC (IE-HPLC), and ion-exchange fast-performance liquid chromatography (IE-FPLC) [107]. Lately, affinity chromatography methods, falling within the category of sorptive techniques, have been increasingly used for RNA preparation. Further, there are examples of successful RNA purification approaches using size-exclusion chromatography (SEC), where the molecules are separated on the basis of molecular size rather than chemical interactions with the stationary phase [106]. All of these techniques and their relevance for RNA sample preparation have been reviewed recently [46, 108–111]. A more detailed description of the different methods follows.

#### Reversed-phase ion-pairing high-performance liquid chromatography

This technique is based on the use of lipophilic cations; quaternary ammonium compounds that ion-pair with the negatively charged sugar–phosphate backbone of the oligonucleotide are commonly used. These ion-paired complexes then become lipophilic and can interact with the stationary phase of a reversed-phase chromatography column (Fig. 4a). The lipophilic oligonucleotide complex, once bound to the column, is eluted and separated with an organic solvent gradient, usually with acetonitrile [107]. As described earlier [108, 109], although RP-IP-HPLC has been used successfully for oligonucleotide separation for almost 40 years for smaller amounts of material (analytical scale), scaling up of the method is rare (preparative scale). Examples of reversed-phase ion-pairing methods, purifying larger amounts of oligonucleotides, in the preparative scale of around 1 mg can be found [112, 113], but for these and similar examples the material purified is exclusively synthetic oligonucleotides, which are already inherently pure. RP-IP-HPLC methods are commonly developed for analytical purposes [114, 115]. Some recent work on the analytical scale might be also applicable to RNA sample preparation: for example, RP-IP-HPLC has been used to analyze and purify double-stranded RNA (dsRNA) from material expressed in *E. coli* [116]. In this work total RNA was extracted from the bacteria and analyzed by RP-IP-HPLC. Advantage was taken of the fact that single-stranded RNA can be subsequently degraded to isolate the dsRNA. This method exemplifies the usability of RP-IP-HPLC to assess the presence and purity of dsRNA as well as the native RNA of the cell (e.g., separation of tRNAs and the different ribosomal RNAs). Another method addresses the separation of stereoisomers to synthetically produce RNAs containing phosphorothioate groups in the backbone by RP-IP-HPLC [117]. This work shows that it is possible to separate stereoisomers using classic reversed-phase ion-pairing chemicals and ion-pairing agents. Triethylamine acetate in combination with the organic modifier acetonitrile was the most successful combination.

#### Ion-exchange high-performance liquid chromatography

Just like the RP-IP-HPLC method, IE-HPLC and IE-FPLC are equally established as a technique for oligonucleotide separation. In this technique the stationary phase already contains the cationic groups for the anionic oligonucleotide to interact and bind with (Fig. 4b). The polymer is then eluted and separated on the column with use of a salt gradient [107]. IE-HPLC has been used successfully within the field of RNA structural biology, and IE-FPLC [109, 118] might be one of the most attractive options to purify milligram amounts of in vitro transcribed RNA. However, even with this method, abortive transcripts can be present in the final purified material for molecules shorter than 30 nucleotides when one starts with a heterogeneous RNA sample. IE-HPLC has also been successfully used together with *trans*-acting hammerhead ribozymes (see “Ribozyme cleavage and T4 ligation”) to purify in vitro transcribed RNA on a milligram scale [62]. The fact that the ribozyme is *trans*-acting can make this approach attractive to purify isotopically labeled RNA samples, since the cleaving ribozyme can be transcribed unlabeled in a completely independent transcription reaction. Several important aspects of IE-HPLC have been reviewed concerning how different cations support different conformers of RNA that subsequently influence the IE-HPLC separation [119].

#### Affinity chromatography techniques

As stated before, the affinity chromatograph technique falls within the category of sorptive techniques; that is, the compound to be separated interacts chemically with the stationary phase (Fig. 4c). The major difference compared with ion-exchange and reversed-phase chromatography is that the mode of interaction between the analyte and the stationary phase is strongly specific and is often inspired by biological interactions [120]. The type of molecules in the stationary phase, the affinity ligands, which interact with the molecule to be separated in the mobile phase, can differ widely: antibodies, proteins, oligonucleotides, dyes, boronate groups, or chelated metal ions are attached to a solid support such as agarose beads or silica material and constitute the stationary phase [121]. This versatility of the stationary phases has led to a number of different RNA purification approaches, some of which were reviewed recently [111]. Unless the RNA to be purified naturally contains a sequence with strong affinity for a target that can be immobilized on the stationary phase, the RNA can be *tagged* with a specific sequence to do so, analogous to the polyhistidine tag used in protein science. There are several ways to achieve this. Affinity tags can be developed by systematic evolution of ligands by exponential enrichment (SELEX) [122]. One approach developed RNA sequences binding streptavidin and Sephadex. The tagged RNA was eluted from the affinity ligand by competitive binding of the natural ligand d-biotin or dextran [123]. In another elegant approach the tag can be combined with compound-activated ribozyme to cleave the product of interest from the stationary phase [124].

#### Size-exclusion chromatography

As the name implies, SEC separation is based on the difference in size or hydrodynamic radius of the molecules. When a porous stationary phase is used, large molecules cannot enter the pores and they pass through the stationary phase, whereas smaller molecules are retained (Fig. 4d). On the basis of this principle, separation is achieved, and the larger molecules are eluted first [106]. SEC systems have been successfully used for preparative-scale RNA sample preparation [125, 126]. Important effects of RNA structure, such as the influence of oligonucleotide secondary structure and its influence on SEC separations, have been investigated [127]. For phosphorothioate oligonucleotides, SEC separation properties have recently been investigated [128]. This work concluded that phosphorothioate oligonucleotides can be efficiently separated by SEC although this is complicated by additional lipophilicity caused by the sulfur atoms in the backbone of the oligonucleotide.

## Conclusions

Sample preparation is currently a bottleneck in the structural characterization of RNA, impeded by RNases and a lack of development for large-scale preparative methods. However, new developments are opening up new avenues that can be combined to answer many new, challenging, and interesting questions in RNA structural biology. Here we reviewed recent progress in well-established production and purification methods that allows preparation of large amounts of RNA commonly needed for structural characterization. Several new exciting methods that are emerging, such as circular RNAs, PLOR, and polymerase chain transcription, have great potential to reduce the amount of work and time needed for RNA sample production. On the other side, obtaining a sample that is pure and homogeneous is usually the most challenging step in structural studies, and new, fast, and innovative methods for sample preparation are currently lacking. However, a combination of typically two different purification methods will most often result in a reliable, easy to work with sample that will save time in the long run.
